# Direct adjusted comparison of expressed emotion towards patients with schizophrenia between halfway houses and family settings

**DOI:** 10.1192/j.eurpsy.2024.608

**Published:** 2024-08-27

**Authors:** P. Ferentinos, S. Douki, E. Kourkouni, D. Dragoumi, N. Smyrnis, A. Douzenis

**Affiliations:** ^1^2nd Department of Psychiatry, “Attikon” University General Hospital, National and Kapodistrian University of Athens; ^2^Department of Psychiatry, Evangelismos” General Hospital; ^3^Center for Clinical Epidemiology and Outcomes research, Athens, Greece

## Abstract

**Introduction:**

Rates of high expressed emotion (EE) towards patients with schizophrenia have only indirectly been compared between families and community residential facilities, since studies including patients in both settings are unfortunately lacking. High EE rates in staff-patient studies are typically lower than in families, with negligible rates of high emotional overinvolvement (EOI). However, indirect comparisons can suffer from many biases.

**Objectives:**

This study directly compared patients with schizophrenia living in halfway houses or with their families on the EE of their caregivers, adjusting for patient- and caregiver-related confounders.

**Methods:**

We included 40 inpatients with schizophrenia living in halfway houses and 40 outpatients living with their families and recorded the EE of the caring staff (N=22 nurses) or parents (N=56), respectively, through Five Minutes Speech Sample interviews. Each nurse rated 1-12 inpatients and each inpatient was rated by 2-5 nurses, totaling 155 nurse ratings. Each outpatient was rated by one or both parents. Due to the multilevel structure of EE ratings, generalized linear mixed models were fitted. We first adjusted only for differences in patient-related confounders between groups and then added basic caregiver-related demographics.

**Results:**

Compared to outpatients, inpatients were older (p=0.001), less well educated (p=0.002), had a longer disease duration (p=0.047), more hospitalizations (p=0.012), lower severity of psychotic (p=0.027) and, specifically, negative symptoms (p=0.015), and lower perceived criticism (p=0.001). Nurses were younger (p<0.001) and better educated (p=0.001) than parents. After adjusting for patient-related confounders only, EOI was significantly higher in parents (p=0.027) while criticism did not significantly differ between groups. However, after also adjusting for caregiver demographics (age, gender and education), criticism was significantly higher in nurses (p=0.027) while differences in EOI became non-significant.

**Conclusions:**

Differences in EE, when directly compared between parents and professional caregivers, may be explained by differences in patient-related characteristics, caregiver demographics as well as other caregiver characteristics to be investigated in future studies.

**Disclosure of Interest:**

None Declared

